# Mr. Grose, on Vaccine

**Published:** 1801-05

**Authors:** J. H. Grose

**Affiliations:** Winslow


### *To the Editors of the Medical and Phyfical Journal.*

Gentlemen,

After the very candid manner in which the utility of Cow-pock inoculation has been inveftigated, it will appear, perhaps, fuperfluous for me to trouble you with any additional obfervations; but as the following fubject has long been the theme of difcuffion, and appears at prefent undecided, it may not be altogether improper to offer a mite of information on a fubject fo interefting to the community.

It is nearly admitted as an axiom, that where puftules have appeared in the Vaccine Inoculation, variolous matter has occafioned them, and there have been fome fo illiberal and injudicious, as to cenfure the new inoculation on this trivial pretext. An extenfive practice has confirmed me in an opinion which I have long adopted, that it is immaterial whether the virus be taken from the incifed part or from another puftule, as the difeafe occafioned by either proves (unlefs fome manifeft caufe to the contrary exifts in the conftitution) equally benign.

It is a point of no fmall importance to afcertain the real caufe of thefe eruptions; and having perufed the publications of various gentlemen who afcribe thefe appearances to the unknown introduction of variola, it becomes a fubject not only curious but interefting, to determine how it is, that matter taken from the teats of the cow, on *a new lancet*, or *penknife*, and inferted with the fame inftrument, fhall produce in five cafes out of fifty,* eruptions from ten to a hundred, &c.

On the other hand, feveral cautious and experienced furgeons have obferved inftances of Cow-pock eruption, when the infection has been taken from the animal and introduced in

---

* Practitioners in general, at prefent, inftead of five cafes out of fifty, would fay, *one case out of a thousand*, fuppofing the Inoculation done properly. Ed.

in the moft precife manner, and have declared themfelves fufficiently fatisfied that they arofe from the introduction of pure Vaccine. I confefs, I know not what to think on the fubject and fhall only relate a few inftances that appear to merit your ferious attention.

Case 1 and 2. Two of the fervants of Mr. Butcher, of Steeple-Claydon, inoculated themfelves with matter from the cow. The ufual appearances fucceeded; they had pain in the axilla, and conftitutionl fever on the eighth day; and on the tenth, one of them had five or fix eruptions on each arm, and a few more on the breaft: the other had five only. Some of thefe eruptions affumed different appearances; for, on one fubject, they were filled with lymph, and others were diftinguifhable by a variolous-like afpect and circular inflammation, and fcabbed on the fourteenth day.

Case 3. The fervant of Mr. Roads, of Claydon, caught the infection by milking, and had the ufual appearances, with nearly twenty puftules on one hand and arm.

Case 4. Mrs. Butcher, of Claydon, inoculated herfelf about three weeks fince, from a girl who had received the difeafe from the cow; and I faw her arm about twelve days after, with a few complete puftules on it.

Case 5 and 6. I inoculated Mr. and Mrs. Verney, of Stewkly, a few months fince, with matter tranfmitted to me by Mr. Lawford of Layton, and both had eruptions.

Case 7. Mr. Turner's maid-fervant, of Winflow, was inoculated the 6th of March, and had in ten days five or fix puftules.

Case 8 and 9. I inoculated two of Mr. Sears's children, of Stewkly; and after, in the courfe of a few days, there was a confiderable eruption on both.

Thefe are plain and important truths, and fuch as muft neceffarily convince the moft incredulous of the non-exiftence of variola, and that thefe appearances are a concomitant of the genuine Cow-pock. I recollect when I faw Mr. Jenner, (nephew to the Doctor) at Tufmore, I related to him one or two inftances; and he obferved, if I am not very much miftaken, that he had witneffed fuch circumftances from a deep infertion of the virus. But there is a material difference between a rafh arifing from cuticular inflammation and eruptions filled with a fpecific virus capable of producing conftitutional difeafe. Neither can they be fuppofed to arife from an inordinate ufe of the matter, or that too much had been introduced, although by many denominated the real caufe; for there muft be a certain quantity of contagion applied to produce conftitutional affection; and it has never been found, either in the inoculation of the Small or Cow Pock, that the feverity of the difeafe depended on the quota inferted, or that puftules were to be attributed buted to this caufe. A fufficiency to produce the difeafe muft be applied, but its fubfequent influence is explicable by very different circumftances. It will not unfrequently happen that a furgeon may inoculate numbers without the leaft appearance of eruption, and yet matter, however cautioufly taken from either of the patients, will in fome produce many eruptions; and it will occafionally occur, that whole families inoculated with Small-pox will experience the diforder in its mildeft form, and vice verfa.

I confefs, unlefs very fubftantial evidence is adduced to prove that eruptions are the effect of variola, (for at prefent nothing more than conjecture has been advanced) I fhall ftill hefitate, and from one very fatisfactory reafon. Allowing that by means unknown, a portion (be it ever fo fmall) of Small-pox virus has been introduced into the fyftem, and that the eruptions are Small-pox eruptions, how comes it to pafs, contrary to all received opinion, that this introduction of variola produces the diforder—*free from contagion?* How can it be reconciled with common fenfe, that a patient inoculated with Small-pox matter, and having puftules, fhall fleep with another, be in conftant fociety, and yet never fpread the difeafe? It cannot be fuppofed, becaufe Vaccine virus is blended with it, that it deftroys contagion, the well known principle of Small-pox; there cannot be an intermediate, for either the difeafe is wholly variolous, or not at all; and if variolous, why not contagious; for if the leaft quota of variola is abforbed, and conftitutional affection fupervenes, no power can prevent infection. Neither in the cafes recited did the appearances on the arms juftify the conclufion, for the incifed part bore the characteriftic marks of pure Vacciola. It is but feldom in this part of the country that, previous to the inoculation for Cow-pox, any courfe of medicine is entered on; and perhaps no plaufible reafon can be affigned why it is omitted. I do not pretend to determine the real caufe of eruptions, I only *know* in the cafes alluded to, the Small-pox ferment never entered the fluids; but I think it not altogether improbable that they may be partly occafioned by certain circumftances of the fkin that determine an undue quantity of the Cow-pock matter either to remain or pafs through, and that purgatives, by diminifhing the determination, may have confiderable influence in preventing their appearance. In fact, it is more confiftent with common obfervation to fuppofe that fanguine plethora, free living, and too great proportion of ftimuli may, in conjunction with caufes hitherto undifcovered, have produced eruptions, than the common conjecture of Small-pox influence.

I am, &c.

